# Eicosanoids and other oxylipins in liver injury, inflammation and liver cancer development

**DOI:** 10.3389/fphys.2023.1098467

**Published:** 2023-02-02

**Authors:** Mario M. Alba, Brandon Ebright, Brittney Hua, Ielyzaveta Slarve, Yiren Zhou, Yunyi Jia, Stan G. Louie, Bangyan L. Stiles

**Affiliations:** ^1^ Pharmacology and Pharmaceutical Sciences, School of Pharmacy, University of Southern California, Los Angeles, CA, Unites States; ^2^ Clinical Pharmacy, School of Pharmacy, University of Southern California, Los Angeles, CA, Unites States; ^3^ Pathology, Keck School of Medicine, University of Southern California, Los Angeles, CA, Unites States

**Keywords:** HCC, liver steatosis, PUFA, eicosanoids, oxylipin

## Abstract

Liver cancer is a malignancy developed from underlying liver disease that encompasses liver injury and metabolic disorders. The progression from these underlying liver disease to cancer is accompanied by chronic inflammatory conditions in which liver macrophages play important roles in orchestrating the inflammatory response. During this process, bioactive lipids produced by hepatocytes and macrophages mediate the inflammatory responses by acting as pro-inflammatory factors, as well as, playing roles in the resolution of inflammation conditions. Here, we review the literature discussing the roles of bioactive lipids in acute and chronic hepatic inflammation and progression to cancer.

## 1 Introduction

Liver cancer consists of primarily hepatocellular carcinoma (HCC) and intrahepatic cholangiocarcinoma (iCCA) with HCC accounting for 75%–85% of all liver cancers (Sung et al., 2021). Virtually all liver cancers are characterized by the presence of inflammation. The majority of HCC emerge in livers with chronic liver diseases that include viral hepatitis and hepatitis caused by alcoholic liver disease (ALD/ASH), and non-alcoholic fatty liver disease (NAFLD/NASH). HBV (Hepatitis B Virus) and HCV (Hepatitis C Virus) infection are associated with 33% and 21% of HCC respectively (Singal et al., 2020). ALD/ASH contributes to 30% of HCC worldwide (Singal et al., 2020), and NAFLD/NASH is estimated to contribute to up to 60% of HCC cases (Sanyal et al., 2010). Recent clinical advances using immune check-point therapy to target inflammation showed promising results for liver cancer treatment (Kudo, 2017; Kubes and Jenne, 2018). Thus, liver inflammation is deemed to play important roles in liver disease progression and cancer development.

We shall discuss the effects of bioactive eicosanoids and oxylipins in liver injury, inflammation and cancer progression with a focus on macrophages, which play important roles in orchestrating liver inflammation and liver disease progression (Tu et al., 2020). Bioactive lipids influence acute and chronic phases of inflammation in a manner that is important for the progression of liver disease. First, during the acute phase of inflammation induced by liver injury, eicosanoids are produced to induce proinflammatory response and mitogenic signal to promote regeneration. Production of pro-resolving oxylipins resolves this inflammation and returns the liver to homeostasis. Second, during sustained liver injury, eicosanoids support chronic inflammation by inducing M2 macrophage polarization but decreased pro-resolving lipids leads to reduced phagocytosis, permitting chronic inflammation. During chronic inflammation, the reprogrammed macrophages orchestrate other immune cell types (not discussed in this review) to establish the tumor microenvironment to promote cancer development. Here, we will first briefly summarize the metabolism of the three major groups of bioactive lipids based on the enzymes that metabolize them. We will also introduce the general biological functions of each group of bioactive lipids. We will then review the literature demonstrating the roles of these lipids on liver inflammation, injury and cancer progression. To further address the role of these lipids in liver disease progression, the last section will focus on their functions in liver macrophage action including polarization and phagocytosis function. Finally, we will provide a summary postulating how these lipids are involved in the progression from liver injury to cancer.

## 2 Overview of bioactive lipids and their functions

**FIGURE 1:**
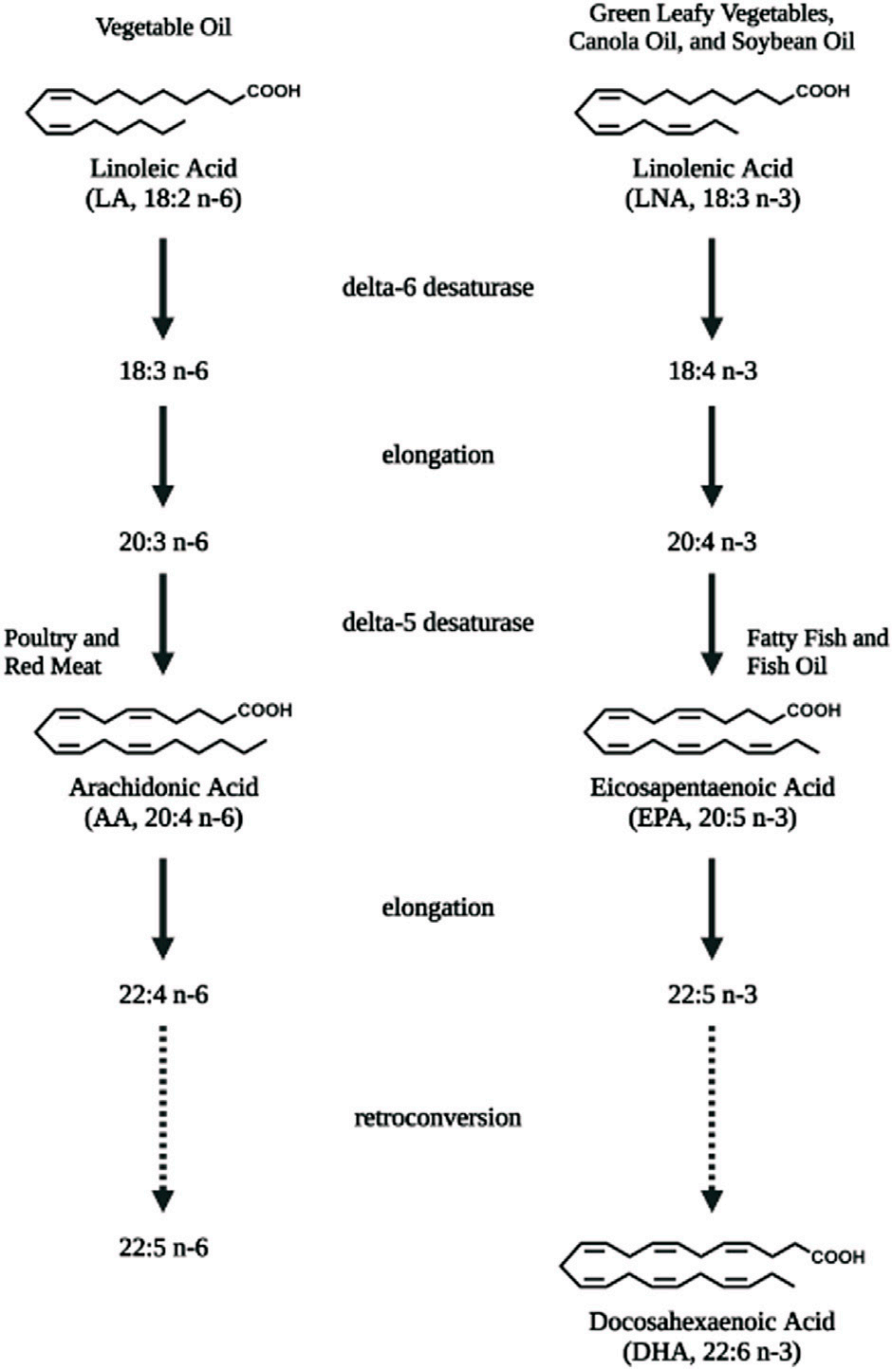
Polyunsaturated fatty acids that serve as substrates for bioactive lipids. Linoleic acid (LA, 18:2 *n*-6), the essential fatty acid of the *n*-6 polyunsaturated fatty acid (PUFA) family is converted to arachidonic acid (AA, 20:4 *n*-6) *via* elongation and desaturation. Linolenic acid (LNA, 18:3 *n*-3), the semi-essential fatty acid of the *n*-3 PUFA family is converted to eicosapentaenoic acid (EPA, 20:5 *n*-3) and longer docosahexaenoic acid (DHA, 22:6 *n*-3) PUFAs *via* elongation, desaturation as well as retro-conversion processes. Diet are rich sources for these PUFAs. Figure created with biorender.com.

**FIGURE 2:**
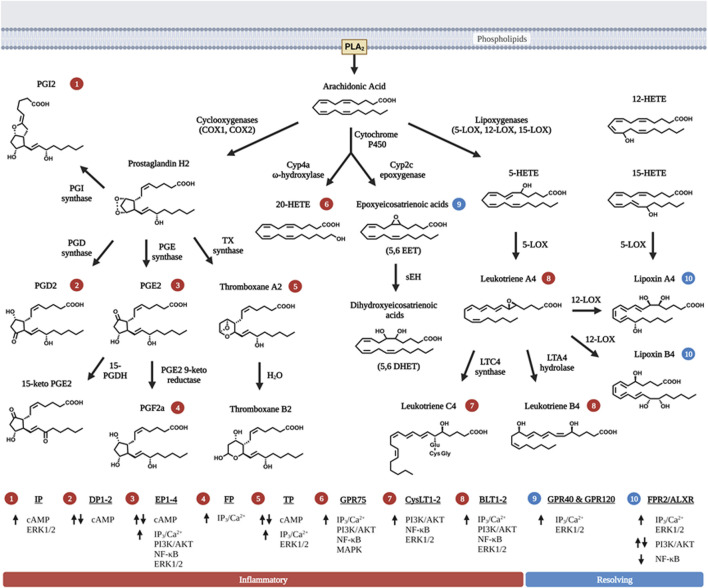
Metabolism of bioactive lipids with arachidonic acid as prototype polyunsaturated fatty acid precursor. Arachidonic acid (AA) is released *via* PLA_2_ and metabolized into one of three distinct enzymatic pathways. First, two isoforms of cyclooxygenase (COX) metabolize AA by forming the signature 5-carbon ring structure observed in prostaglandins (PGH2, PGD2 and PGE2). The intermediate metabolite PGH2 is further metabolized to PGD2, PGE2, PGI2, or TXA2 *via* the action of specific enzymes that leading to their synthesis. PGE2 and TXA2 (thromboxane A2) are further hydrolyzed to PGF2a and TXB2. Second, three primary forms of lipoxygenase (LOX) acts on AA to produce leukotrienes (LTA4, LTB4 and LTC4). 12-LOX also act upon the primary leukotriene, LTA4 to produce lipoxins (LXA4 and LXB4), which have pro-resolving function towards inflammation, rather than pro-inflammation. 15-LOX action can also lead to the production of LXA4 and LXB4. Third, two groups of cytochrome P450 (Cyp2 and Cyp4) act upon AA to produce a series of HETE, EET and DHET products with 20-HETE being the most abundant and best characterized member of these metabolites. Like lipoxins, EETs also possess pro-resolving properties but they are relatively shorter half-life. Non-enzymatic reactions that produce eicosanoids-like compounds are not shown here in the figure. The numbers in circle—indicate receptors that the respective eicosanoids uses to signal. The receptors corresponding to the circled numbers are listed at the bottom of the figures with their respective signals. Up arrows are indicative of activation, and down arrows of downregulation of expression. Eicosanoids with red circled numbers have general functions towards inflammation whereas those with blue circled numbers are generally pro-resolving towards inflammation. Some eicosanoids play a dual role in both activation and inhibition of signaling cascades. Figure created with biorender.com.

Lipids are important energy sources and also serve as essential nutrients needed for the maintenance of membrane structure and integrity in addition to other functions. Polyunsaturated fatty acids (PUFAs) (Figure 1), particularly the *n*-6 family of linoleic acid (18:2 *n*-6, LA), gamma linolenic acid (18:3 *n*-6, γ-LNA)and arachidonic acid (20:4 *n*-6, AA) together with the *n*-3 family of alpha linolenic (18:3 *n*-3, α-LNA), eicosapentaenoic acid (20:5 *n*-3, EPA) and docosahexaenoic acid (22:6 *n*-3, DHA) serve as substrates for the production of bioactive lipids that mediate inflammatory responses (Saini and Keum, 2018). These PUFAs are primarily oxidized by three sets of enzymes that act on different carbon positions of the acyl chain to produce a variety of bioactive oxidized lipids known as oxylipins. Figure 2 depicts these three enzymatic processes using AA (20:4, *n*-6) as the prototype substrate.

### 2.1 Enzymatic pathways for the biosynthesis of oxylipins

Among the enzymes, cyclooxygenases (COX1&2 encoded by PTHS genes) are the most studied (Lambert et al., 1987; Dubois et al., 1998; Kawahara et al., 2015). The COX enzymes add two oxygens to the acyl chains of PUFA, leading to the formation of prostanoids with the 5-carbon member ring structure and the endoperoxide bridge from which products of COX are derived. These products include the prostaglandins (D, E, F, G, H, and I) and thromboxane (TXA and TXB) where those produced from AA are designated with 2-series and those produced from EPA are designated with 3-series. The actions of lipoxygenases (5-LOX, 12-LOX, 15-LOX and LOXE3 encoded by corresponding ALOX genes) produce leukotrienes (LTs), so named because they were originally isolated from leukocytes (Mashima and Okuyama, 2015; Kulkarni et al., 2021). These are products of oxidation and epoxidation reactions occurring on different positions of the acyl chain. These products (LTA, LTB, LTC, LTD and LTE) are designated with 4-series from AA and 5-series from EPA. In addition, intermediate products such as hydroperoxyl- and hydroxy-eicosatetraenoic acids (HETEs) that are also biologically active themselves.

Metabolites due to the pleiotropic effect of cytochrome p450 monooxygenase (CYP) activity are added to the family of bioactive lipids with omega hydroxylation primarily carried out by the CYP4 isoform subset, and epoxygenation by CYP2 isoforms of the CYP enzymes (Panigrahy et al., 2010). As the biological functions of these intermediates and derivatives are discovered after their structure, they are often named based on their chemical structures. For example, EETs (epoxyeicosatrienoic acid, 5,6-EET, 8,9-EET, 11,12-EET, and 14,15-EET) with the numbers designating the position of the epoxy groups are epoxygenase products of AA due primarily to the action of CYP2C and 2J whereas those produced from EPA are referred to as EEQs or EpETEs (epoxyeicosatetraenoic acids) (Panigrahy et al., 2010). The biologically active EET metabolites are then metabolized further *via* soluble epoxide hydrolase (sEH) to dihydroxyeicosatrienoic acids (DHETs) (Yu et al., 2000). Another group of Cyp enzymes, Cyp4s with ω-hydroxylase activity produces HETEs (16-HETE, 17-HETE, 18-HETE, 19-HETE and 20-HETE) from AA. These Cyp enzymes also act on EPA and produce HEPEs (hydroxy eicosapentaenoic acids) which have one more double bond than corresponding HETEs. The microsomal Cyp enzymes also react with AA to produce HETEs *via* an intermediate hydroperoxy-compound similar to the reaction of LOX. Many of these derivatives are not stable and are rapidly converted to other products *via* both enzymatic and non-enzymatic actions. Finally, non-enzymatic oxidation of PUFAs also produces metabolites that are structurally related to the enzymatic metabolites (Milne et al., 2008; Austin Pickens et al., 2019).

### 2.2 Proinflammatory roles of eicosanoids and eicosanoids-like bioactive lipids

The PGs, TXs and LTs, the better-known bioactive lipids are referred to as eicosanoids. Many of these were first discovered as important mediators for inflammatory cell/cardiovascular system functions prior to the discovery of their substrate precursors. The general functions of these classical eicosanoids are summarized in Table 1 and have been extensively reviewed. Typically, EPA-derived 3-series PGs/TXs and 5-series LTs have milder effect compared to those produced from AA, and thus are the preferred inflammatory mediators.

**TABLE 1:** Examples of Eicosanoid’s function in the liver.

Eicosanoid	Primary liver cells of interaction	Biological implication
PGE_2_, PGD_2_, PGF2_a_	Hepatocytes, Kupffer	Enhanced proliferation, vasodilation, increased immune infiltration, proinflammatory
TXA_2_	Kupffer, Stellate	Vasoconstrictor, increased immune infiltration, proinflammatory, enhanced fibrosis
LTA_4_, LTB_4_, LTC_4_	Kupffer, Hepatocytes	Proinflammatory, increased immune infiltration
LXA_4_, LXB_4_	Kupffer, Hepatocytes	Pro-resolving, enhanced efferocytosis, decreased immune infiltration
12-HETE, 20-HETE	Hepatocytes, Kupffer	Proinflammatory, increased immune infiltration, angiogenesis, vasoconstrictor
5,6-EET, 11,12-EET	Sinusoidal endothelial	Pro-resolving, enhanced proliferation, angiogenesis
RvE1, RvD1, MaR1	Kupffer, Hepatocytes	Pro-resolving, decreased immune infiltration, decreased fibrosis

The functions of the non-classical eicosanoids are less characterized. These include the pro-resolving eicosanoids produced from EPA and DHA as well as the EETs and HETEs produced from AA. Similar to PGs, TXs and LTs, HETEs produced from AA display a pro-inflammatory role in general. For example, 20-HETE, one of the best characterize HETEs, is considered to be pro-inflammatory as it mediates the effects of angiotensin II (AngII) on vasoconstriction and other vascular and renal functions (Savas et al., 2016). Treatment of human endothelial cells with 20-HETE resulted in the induction of inflammatory cytokines including IL-4, IL-8, and IL-13 (Ishizuka et al., 2008). Systemic inflammatory response is also associated with the induction of 12-LOX in macrophages and 12-HETE has been found to induce the expression of pro-inflammatory cytokines in cultured adipocytes (Chakrabarti et al., 2009). The AA-derived 5-HETE *via* 5-LOX is further metabolites to LTs and therefore displays proinflammatory functions (Enyedi et al., 2013).

### 2.3 Pro-resolving and anti-inflammatory roles of eicosanoids and eicosanoids-like bioactive lipids

In the presence of 12-LOX, 5-HETEs can also be converted to lipoxins and their derivatives that possess anti-inflammatory functions (Figure 2). Lipoxins were first discovered from leukocytes treated with calcium ionophore (Serhan et al., 1984). They elicit the generation of oxygen particles from neutrophil without inducing elastase release from the lysosome. Subsequent studies established lipoxin to have both anti-inflammation and pro-resolving functions of inflammation *via* inducing phagocytosis of apoptotic neutrophils by macrophages (Godson et al., 2000; Maderna and Godson, 2009). In addition, EETs produced *via* Cyp2 enzymes are also anti-inflammatory as they attenuate VCAM expression in endothelial cells and inhibit macrophages secretion of cytokines (Fleming, 2014).

**FIGURE 3:**
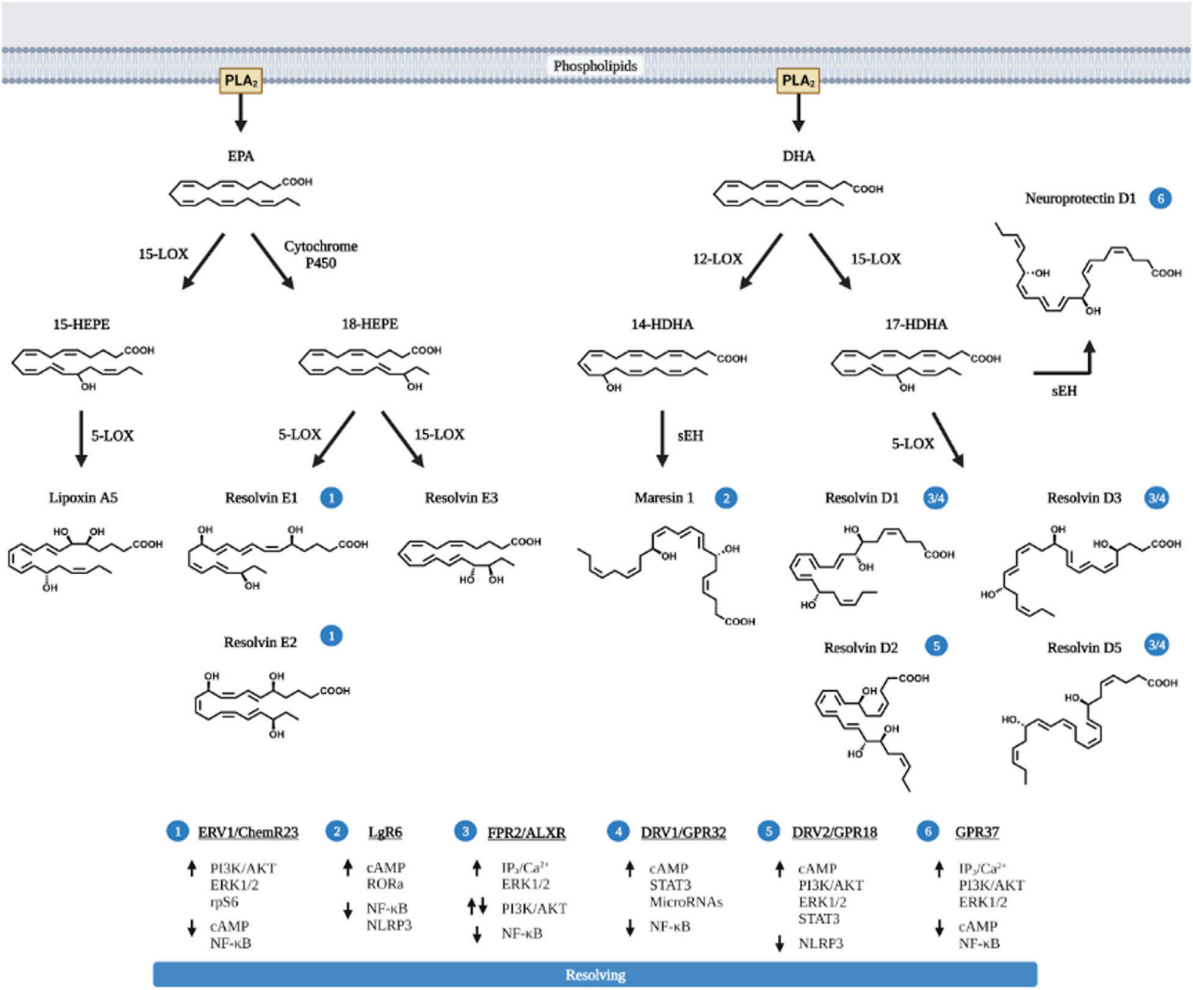
Pro-resolving lipids metabolized from EPA and DHA. EPA and DHA are the primary *n*-3 PUFA substrated of eicosanoid metabolites and are released *via* PLA_2_. Like AA, EPA and DHA are also metabolized by COX and LOX to produce the pro-inflammatory eicosanoids (prostaglandins, thromboxanes and leukotriens, not shown in the figure) with less potency compared to those produced from AA. In addition, EPA and particularly DHA produces pro-resolving metabolites. These pro-resolving eicosanoids (lipoxin A5, LXA5) are produced *via* the action of 12-LOX, similar to LXA4 produced by 15-LOX; or *via* CYP450 to produce Resolvin E (RvE1, E2 and E3) from EPA. The action of 12-LOX and 15-LOX on DHA produces not only resolvins (RvD1, D2, D3, and D5) but also neuroprotectin D1 and Maresins (MaR). The cellular receptors for each pro-resolving eicosanoids and their respective signaling pathway are presented in the bottom of the figure and labeled with circled numbers—near the metabolites. Up arrows indicate enhanced activation, down arrows are indicative of downregulation of signaling pathway. Figure created with biorender.com.

Beyond AA, the extra double bonds in EPA and DHA derived metabolites present unique anti-inflammatory and also pro-resolving functions (Ishihara et al., 2019). HEPEs produced from EPA and HDHAs (hydroxy docosahexaenoic acids) produced from DHA possess pro-resolving functions towards inflammation (Figure 3). These products, also referred to as specific pro-resolving metabolites (SPM) include lipoxins (LXA5 produced from EPA, and also LXA4 from AA) and also resolvins (RvD and RvE), maresins (MaR) and neuroprotectins (NPD).

### 2.4 Receptor mediated signals of bioactive oxylipins

Once released from the source cells, these lipids bind to cell surface receptors that are G-protein coupled receptors (GPCR) on surrounding cells *via* Gs-coupled cAMP release (Figure 2). PGE2 binds to PGE receptors EP1, EP2, EP3, and EP4 in a concentration dependent manner (Kalinski, 2012). EP1 and EP2 stimulation requires higher PGE2 concentrations to initiate the signaling cascade whereas EP3 and EP4 are stimulated with lower PGE2 concentrations. Stimulation of the EP2 and EP4 receptors then activate ERK1/2, AKT, NFκβ, and β-catenin signaling pathways to improve cell survival and motility (Banu et al., 2009). Other prostanoids also have their own specific receptors and signaling pathways (Figure 2). The biological response to these prostanoids is dependent on the receptors/signaling pathways (Shapiro et al., 2016).

Mechanistically, resolvins (RvD, E and MaRs) binds to the same receptor (ALXR) that LXA4 acts on (29). Originally identified as low-affinity N-formyl-methionyl-leucyl-phenylalanine receptor-like-1 (FPR1), ALXR also binds to other pleiotropic ligands including RvD/E and MaRs (Scannell and Maderna, 2006). The actions of EETs and HETEs also requires G protein coupled receptors. Several EET and HETE receptors have been documented including GPR75, GPR40, and GPR120 (Wong et al., 1993; Yang et al., 2008; Nguyen et al., 2016; Garcia et al., 2017; Park et al., 2018).

## 3 Biological functions of oxylipins in liver disease and cancer progression

Bioactive lipids have been identified as important mediators for the progression of liver disease and cancer. In HBV-cirrhosis and HCC patients, 42 and 31 PUFA metabolites, respectively, were found to be significantly altered (Gong et al., 2017). In mouse models of HCC, distinct eicosanoid serum profiles also distinguish between HCC and normal control mice (Li et al., 2018). Here, we will discuss the literature demonstrating the involvement of eicosanoids/oxylipin and their metabolizing enzymes in the progression of liver disease and cancer development.

### 3.1 COX and PGE2 in liver disease and cancer progression

**FIGURE 4:**
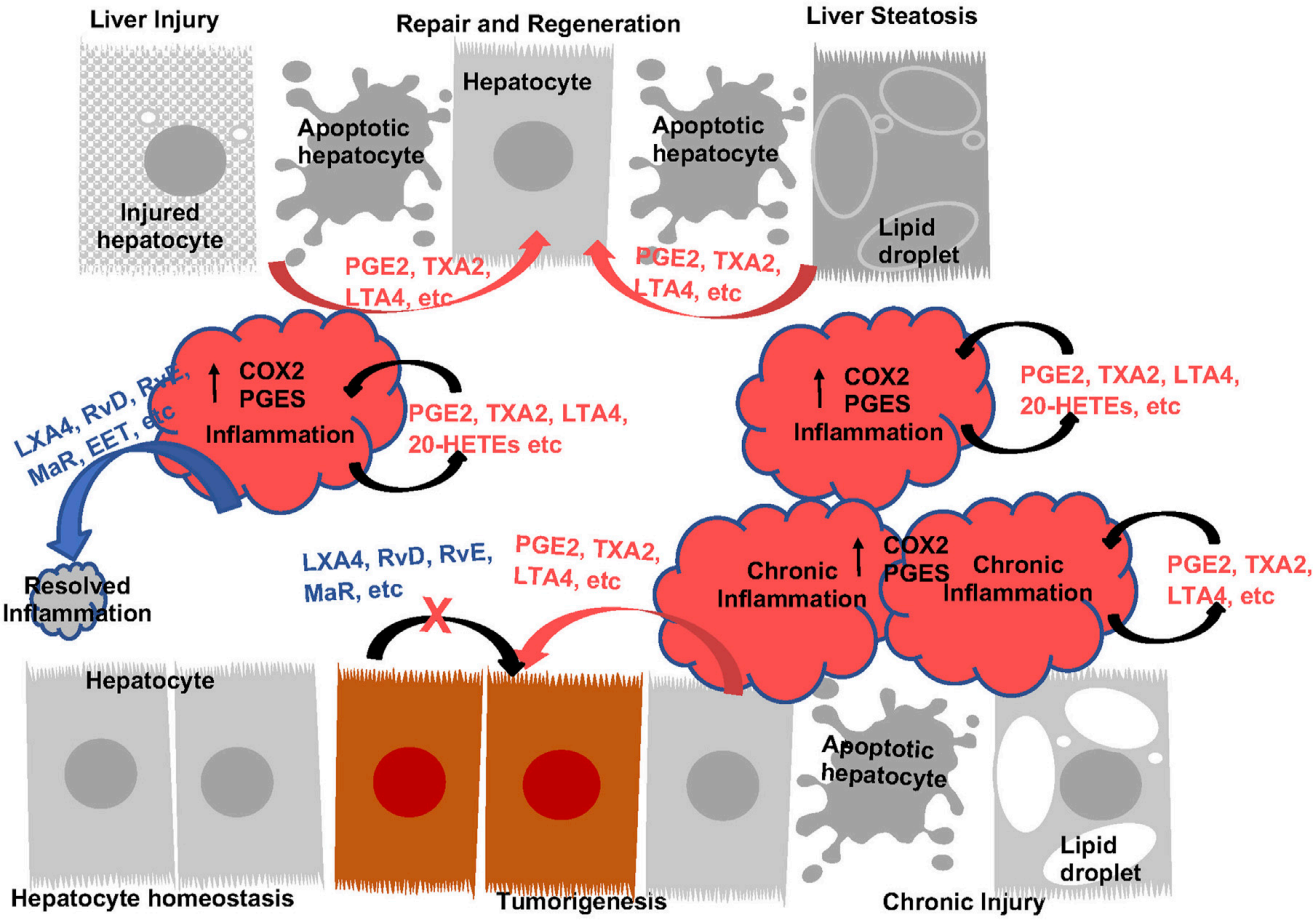
The Function of Eicosanoids and Oxylipins in Liver Inflammation and Liver Disease Progression. During acute inflammatory response, prostanoids (PGE2 an TXA2, etc.) and leukotrienes (LTA4, etc.) are induced to induce inflammation. These eicosanoids plays roles to sustain inflammatory state as well as induce regeneration by acting as mitogenic signals for hepatocytes. During this process, induction of pro-resolving eicosanoids (LXA4, RvD, RvE, MaR, etc.) resolves inflammation, return the liver to homeostasis. With sustained injury, induction of eicosanoids sustains inflammation and establishes an environment for tumor growth. These eicosanoids also acts as mitogenic signals to promote tumor cells growth. The lack of pro-resolving lipids contributes to the chronic inflammatory state. Addition of pro-resolving eicosanoids also directly attenuate tumor cell growth.

The naive liver expresses high levels of COX1 as well as downstream enzymes whereas the expression of COX2 is low (Koga et al., 1999; Bezugla et al., 2006). LPS induction leads to the upregulation of COX2 concurrent with PGE2 synthase 1 (PGES1) in liver resident macrophages, the Kupffer cells (Bezugla et al., 2006). Consistently, LPS induces the robust production of PGE2 and TXA2 in the liver (Bowers et al., 1985; Bezugla et al., 2006; Miller et al., 2007; Miao et al., 2016; Zhang et al., 2020a). Thus, the induction of COX2 appears to contribute to the inflammatory response and is also considered to have generally cytoprotective effects that are attributed primarily to PGE2 (Figure 4).

#### 3.1.1 COX2, PGE2 and liver injury

The pro-inflammatory role of COX2 and PGE2 in the liver are illustrated by studies using diet to induce chronic inflammation or bacterial toxins/physical injury to induce acute inflammation (Figure 4). During chronic liver injury conditions, such as NAFLD and NASH, COX2 metabolism is implicated where both COX1 and COX2 expressions are induced (Lampiasi et al., 2006; Henkel et al., 2018; Garcia-Jaramillo et al., 2019; Sztolsztener et al., 2020). EPA feeding inhibits NAFLD (Ishii et al., 2009; He et al., 2010; Albracht-Schulte et al., 2019; Chen et al., 2021a) suggesting that AA-derived eicosanoids play a role in the development of NAFLD. Consistently, interventions that reduce NAFLD is associated with decreased PGE2 in circulation (Cansancao et al., 2020; Maciejewska-Markiewicz et al., 2022). Furthermore, pharmacological inhibition of COX2 with celecoxib attenuates hepatic steatosis and associated inflammation (Chen et al., 2011; Wu et al., 2016; Liu et al., 2018; Zhang et al., 2022). AKT kinase, NFκβ and autophagy are implicated in this role of COX2. In addition, deficiency of PGES2 also led to reduced liver injury and inflammation in Methionine-choline deficient (MCD) diet induced NASH (Zhong et al., 2022), suggesting that PGE2 maybe driving the effects of COX2. In response to acute injury, liver also upregulates the expression of COX2 and production of PGEs and TXs (Bowers et al., 1985; Reilly et al., 2001; Bezugla et al., 2006; Miller et al., 2007; Shimada et al., 2020). COX2 inhibition resulted in reduced neutrophil infiltration and protection against ischemia reperfusion (I/R) injury (Kuzumoto et al., 2005; Ozturk et al., 2006; Tolba et al., 2014). In LPS/Galactosamine (GalN) induced acute liver injury model, decreasing PGE2 with hepatocyte-targeted expression of 15-PGDH led to less hepatic apoptosis/necrosis (Yao et al., 2017). Thus, COX2 and its metabolites indeed play a proinflammatory role during both acute and chronic liver injury (Figure 4).

While COX2 and PGE2 induce inflammation, suggesting that they might promote further liver injury, genetic studies have also demonstrated a hepatoprotective effects for these lipids. This cytoprotective effects is responsible for hepatocyte regeneration after injury (Figure 4). Using the apolipoprotein E (ApoE) promoter to drive the expression of COX-2 led to protection against diet-induced liver steatosis (Frances et al., 2015). Consistent with this result, global loss of PGES1 augmented hepatocyte apoptosis and increased liver inflammation, particularly TNFα releases when treated with LPS (Henkel et al., 2018). As COX enzymes and PGE2 are also expressed in liver hepatocytes, these genetic studies together suggest cell- and context-specific functions of COX2-PGE2 axis in liver injury. Supporting this notion, deletion of COX2, treatment with celecoxib or CAY10526, a PGES inhibitor result in more severe toxicity/lethality induced by overdose of acetaminophen (Reilly et al., 2001; Cavar et al., 2010). Thus, the mitogenic role of PGE2 on inducing hepatocyte proliferation may have contributed to this “cytoprotection” effect of PGE2 and COX2 (Bowers et al., 1985; Tsujii et al., 1993) despite of their proinflammatory function. However, some recent data also suggests an anti-inflammatory properties of PGE2 in the cardiovascular system (Gitlin and Loftin, 2009; Kirkby et al., 2014) that might be receptor isoform mediated (Takayama et al., 2002; Tang et al., 2012). In the liver, this is supported by the accelerated development of NASH in MCD diet fed mice lacking PGI2 receptor (Kumei et al., 2018) and reduced neutrophil infiltration and protection against I/R injury in EP4 agonist treated mice (Kuzumoto et al., 2005). Therefore, a balanced pro- and anti-inflammatory function of COX2/PGE2 and their role towards cell growth and apoptosis during liver disease progression likely determine their roles at given stages of the disease (Figure 4).

#### 3.1.2 COX2, PGE2 and progression to liver cancer

Overexpression of COX-2 and PGE2 receptors is observed in most tumor types, including HCC (Koga et al., 1999; Kondo et al., 1999; Shiota et al., 1999; Sung et al., 2004; Breinig et al., 2008; Zang et al., 2017) and is further induced when coupled with HFD (Koga et al., 1999; Hamzawy et al., 2015). Expressions of COX2 also correlates with the differentiation status of liver cancer (Koga et al., 1999; Kondo et al., 1999; Shiota et al., 1999; Sung et al., 2004) where high COX-2 expression is associated with lymph vascular invasion and distant metastasis with poor 5-year survival (Tai et al., 2019). Supporting a pro-tumor role of COX-2 in HCC development, knockdown of COX-2 resulted in reduced cell proliferation and significantly decreased colony formation in cultured HCC cell lines (Austin Pickens et al., 2019). Expression of COX2 in cultured hepatocytes also suppressed caspase activity concurrent with reduced p53 and Bax expression, suggesting a role of COX-2 in apoptosis as well (Fernandez-Martinez et al., 2006). Consistently, ectopic expression of COX-2 in the livers of transgenic mice was sufficient to induce spontaneous HCC development (Chen et al., 2017), though other studies show preneoplastic lesions with minor contribution to the malignant transformation to HCC (Llorente Izquierdo et al., 2011). Further, inhibition of COX-2 attenuates HCC growth in animal models (Hamzawy et al., 2015; Ali et al., 2022) and celecoxib dose-dependently reduce tumor weight (Cui et al., 2005; Li et al., 2016). These genetic and pharmacological studies indicate a pro-tumor role of COX-2 and its metabolite in liver cancer progression (Figure 4).

As the major product of the COX2-mediated metabolites of AA(26), elevated serum PGE2 levels are associated with larger HCC tumor sizes and poor overall survival (Gong et al., 2017; Li et al., 2018; Tai et al., 2019; Pelizzaro et al., 2021). Transgenic expression of HBV X protein (HBx) that promotes tumor development also leads to increased PGE2 in the serum (Lan et al., 2022). In HCC cells, PGE2 level is associated with enhanced cell proliferation and invasion due to upregulated expression of survivin (Bai et al., 2010), c-Myc (Xia et al., 2014), and β1-integrin (Bai et al., 2014). The mitogenic effects of PGE2 has been found to be mediated *via* EP3 receptor in cultured rat hepatocytes in which PGE2 dose- and time-dependently induced DNA synthesis (Hashimoto et al., 1997). A role of PGE2 on inducing tumor cell invasion and migration has also been reported (Mayoral et al., 2005; Bai et al., 2009; Zhang et al., 2014a; Zhang et al., 2014b; Cheng et al., 2014; Xia et al., 2014). Consistent with a promo-tumor growth role of PGE2, genetic studies showed that overexpression of 15-PGDH suppresses while its knockdown induces the growth of HCC cells and tumor grafts (Lu et al., 2014). An opposite effect is observed when PGES1 is targeted (Lu et al., 2012).

The involvement of serine/threonine kinase AKT and mTOR in COX-2 regulated pro-tumor effects have been reported (Leng et al., 2003; Liu et al., 2005; Qiu et al., 2019; Tai et al., 2019). In steatotic livers, celecoxib blocks insulin regulated lipid accumulation *via* its actions on AKT (Lu et al., 2016), which has been shown to drive *de novo* lipogenesis (He et al., 2010; Li et al., 2013; Palian et al., 2014; Chen et al., 2021a). COX-2 also regulates apoptosis and cell proliferation in HCC *via* AKT signaling as dephosphorylation of AKT is observed concurrent with induction of cell death and reduced PCNA staining (Leng et al., 2003; Liu et al., 2005). The regulation of tumor suppressor PTEN, a negative regulator for AKT-mTOR signal (Tu et al., 2020), and its effect on tumor progenitor cells may play a role in the effect of COX-2 on hepatic tumorigenesis (Rountree et al., 2009; Galicia et al., 2010; Chu et al., 2014; Guo et al., 2015; Debebe et al., 2017; Chen et al., 2021b). Other signals involved in PGE2 regulated cell growth and survival includes growth factor signals (Koide et al., 2004; Fernandez-Martinez et al., 2006; Odegard et al., 2012; Tveteraas et al., 2012; Zhang et al., 2014a), mitochondrial function (Kern et al., 2006), ER stress signal (Lee et al., 2020; Su et al., 2020) as well as the HIF-1α pathway (Dong et al., 2018). A synergistic effect has been observed for sorafenib, the multikinase inhibitor used as first-line therapy for HCC and celecoxib (Cervello et al., 2013).

### 3.2 Thromboxanes, leukotrienes and proinflammatory oxylipin in liver disease

Compared to PGE2 and COX2, significantly less is understood regarding the roles of other eicosanoids/oxylipins in liver disease progression. In general, other eicosanoids and the proinflammatory oxylipins exhibited similar effects towards hepatoprotection/proliferation and tumor growth (Figure 4). Like PGE2 and COX2, these effects are cell type specific and the functional outcome is dependent on the cell types which the manipulations are targeted in a given experiment (Figure 4).

#### 3.2.1 Thromboxanes

Thromboxanes regulate liver micro vasoconstriction functions and stimulates the release of proinflammatory cytokines that impacts platelets and recruitment of leukocytes (Iwata, 1994; Yokoyama et al., 2005). In response to LPS stimulation, TXA2 and its stable metabolite TXB2 are released from macrophages prior to PGE2, likely due to the expression of TXA2 synthase that is already present in the naïve livers (Bowers et al., 1985; Bezugla et al., 2006; Miller et al., 2007). Elevated plasma thromboxane is found to correlate with the severity of liver injury (Nanji et al., 1993; Shimada et al., 1994; Suehiro et al., 1996; Nanji et al., 1997). Treatment with TX receptor antagonist or TXA synthase inhibitor prevents the necroinflammatory changes, reduced injury and also reduces alcohol feeding induced fibrosis (Suehiro et al., 1996; Nanji et al., 1997; Ito et al., 2003; Nanji et al., 2013). In addition, reducing levels of TXA2/B2s is implicated in statin and riboflavin mediated suppressive effects on NASH induced injuries (Ajamieh et al., 2012; Wang et al., 2018). Despite this pro-inflammation/injury role, TX signal is also necessary for promoting liver regeneration. Deletion of TXA2 receptor TP or treatment with TXA2 inhibitor results in impaired ability for mice to recover from partial hepatectomy (PHx) or carbon tetrachloride (CCL4) induced injury with elevated necrosis and delayed hepatocyte proliferation (Minamino et al., 2012; Mohamed et al., 2020). Thus, similar to that of PGE2, the mitogenic function of TXA2 *versus* its effects towards inflammatory response determines whether TXA2 has a hepatoprotective or pro-injury/inflammation effects in any given experimental condition (Figure 4). Non-etheless, these observations indeed implicate a pro-tumorigenic role of TXA2 in modulating the tumor immune environment as well as tumor growth, though experimental data are still needed to specifically address this function of TXA2 in liver cancer. Of note, in a model of colon cancer metastasis to the liver, inhibiting TXA2 synthase showed more significant inhibition than aspirin (Yokoyama et al., 1995), suggesting that the TXA2 indeed supports a pro-tumor microenvironment.

#### 3.2.2 Lipoxygenase, leukotrienes and proinflammatory oxylipins

In HCC patients, elevated leukotriene metabolites is reported (Zhou et al., 2011). Experimental evidence demonstrates that 5-LOX expression is increased in several rodent models of liver disease, including liver fibrosis induced by CCl_4_ and MCD diet (Pu et al., 2021), acetaminophen-induced liver injury (Pu et al., 2016), diethyl nitrosamine (DEN)-induced HCC (Xu et al., 2011), HFD-induced NAFLD/NASH (Ma et al., 2017), and hepatic steatosis due to ApoE deficiency (Martinez-Clemente et al., 2010). Accordingly, inhibition or loss of 5-LOX attenuates or protects the mice against these conditions (Martinez-Clemente et al., 2010; Hohmann et al., 2013; Pu et al., 2016; Ma et al., 2017; Pu et al., 2021). Mechanistically, 5-LOX and its metabolite LTB4 is found to activate NF-Kβ in HCC cells (Zhao et al., 2012). Inhibition of 5-LOX and LTB4 resulted in decreased PCNA and cyclin D expression after HPx (Lorenzetti et al., 2019). A positive feedback loop for 5-LOX and FASn is identified that involves LTB4 (Chiu et al., 2019). Production of another metabolite of LOX, 5-HETE is also perturbed in the DDC induced liver injury model (Pandey et al., 2014). Reducing 5-HETE leads to mitigation of arsenic-induced NASH (Wei et al., 2020). These studies together support a pro-inflammatory role of 5-LOX and its metabolites during liver injury and a pro-growth role on hepatocytes (Figure 4).

Similar functions in liver disease and cancer have been reported for 12-LOX and 15-LOX (Tanaka et al., 2012; Xu et al., 2012; Ma et al., 2013; Yang et al., 2019). In HCV-HCC patient samples, both 12-HETE and 15-HETE are found elevated (Fitian et al., 2014). Deficiency of 12-LOX and 15-LOX activity attenuated steatosis, liver injury and inflammations observed in *ApoE* ^*−/−*^ mice (Martinez-Clemente et al., 2010). 12-HETE, the product of 12-LOX is found to be increased in plasma of NASH mice induced by MCD diet (Tanaka et al., 2012). Inhibiting 12-LOX activity attenuates HCC tumor cell growth and inhibits HFD promoted HCC development (Xu et al., 2012; Yang et al., 2019). 15-HETE production is disturbed in DDC treated mouse livers (Pandey et al., 2014). Reducing 15-HETE *via* inhibiting 15-LOX results in apoptosis (Ma et al., 2013). Similar to that of COX enzymes, PI3K-AKT signals are proposed to be involved in the cell survival/proliferation regulation by 12- and 15- LOX (Ma et al., 2013; Yang et al., 2019).

#### 3.2.3 Cyp450 and proinflammatory oxylipin

CYP enzymes are highly expressed in the liver. These enzymes are responsible for the vast majority of drug metabolism, but also play a significant role in xenobiotic elimination, where their dysfunction can lead to underlying liver diseases (Mukkavilli et al., 2014; Shoieb et al., 2020). Previous studies have established that etiologies such as ALD and cirrhosis have isoform specificities in their impact on CYP metabolism (Marino et al., 1998; Yang et al., 2003) with CYP2C and CYP2D being the most altered between healthy controls and liver disease cohorts (Frye et al., 2006). In HCC, Cyp enzymes responsible for PUFA metabolism have been identified among the top genes enriched in a study aimed at elucidating prognostic markers (Ding et al., 2022). This study also identified CYP26A1, CYP2C9 and CYP4F2 among a proposed prognostic panel of genes when trying to model the risks for iCCA and HCC (Ding et al., 2022). In a separate cohort, the expression of CYP2A6 was also closely associated with tumor grades and favorable prognosis (Jiang et al., 2021). In addition, several CYP4 enzymes are found to correlate with favorable outcomes for HCC and their protein expressions have been verified using immunohistochemical staining (Eun et al., 2018). Products of Cyp, such as 14,15-DHET have been found to correlate with liver cancer diagnosis marker alpha fetoprotein (AFP) in HBV-related HCC patient samples (Lu et al., 2018a) and NASH/fibrosis (Caussy et al., 2020). In cultured Huh7 cells, introduction of HCV core protein NS5A alters the expression of CYP2E1 (Smirnova et al., 2016). Together, these studies suggest that certain Cyp450 regulated oxylipins also play a role in promoting liver disease progression and cancer development.

Of the CYP produced oxylipins, 20-HETE is by far the most abundantly produced, accounting for 50%–75% of all Cyp450 eicosanoids produced in the liver and as such is one of the most characterized Cyp450 eicosanoids (Sacerdoti et al., 2003a). Analysis of cirrhosis cohorts has revealed elevated levels of 20-HETE as the predominant eicosanoids, even higher than that of PGs and TXs demonstrating the potential significance these eicosanoids have in liver disease progression (Sacerdoti et al., 1997; Li et al., 2023). Plasma levels of 20-HETE are also increased in NAFLD and alcoholic liver disease (ALD) patients among other proinflammatory oxylipins including 12-HETE and 8-HETE (Gao et al., 2019; Li et al., 2020a). In experimental models, 20-HETE has been shown to induce the activation of LX-2 cells *via* TGFβ signaling through proteasome regulation (Lai et al., 2018; Li et al., 2023) and inhibiting 20-HETE production attenuates liver fibrosis induced with CCL4 (Li et al., 2023). This effect may involve ubiquitination as 20-HETE decreases the expression of Nedd4-2 in the liver (Zhao et al., 2017).

### 3.3 Pro-resolving and anti-inflammatory eicosanoids and liver disease

In addition to the proinflammatory metabolites, the SPMs counteract these effects in the liver (Figure 4). A univariate analysis revealed that the recurrence-free survival rate was significantly lower in patients with higher mPGES-1 level in non-cancerous liver tissue (Nonaka et al., 2010). On the other hand, higher expression of CYP4F2 in non-neoplastic liver tissues is associated with a less severe pathological tumor stage (Eun et al., 2018).

#### 3.3.1 AA metabolite EETs produced *via* Cyp450

Correlation studies have shown that EET levels are inversely correlated with NAFLD severity (Arvind et al., 2020). As steatosis progresses to fibrosis, epoxygenase activity significantly declines resulting in decreased EET levels. The oxylipin11,12-EET ameliorates free fatty acid induced inflammation through inhibition of NFκβ signaling in liver macrophages (Wang et al., 2019). The effect of biologically active EETs on vasoconstriction and inflammation maybe dependent on its ability to counteract that of 20-HETE which induces these effects (Lasker et al., 2000; Sacerdoti et al., 2003b). In experimental models, LPS decreases EET while increases 20-HETE (Anwar-mohamed et al., 2010; Theken et al., 2011). The importance of the 20-HETE/EET ratio is supported by studies using CYP4F2 transgenic mice (Zhang et al., 2016). In HCC, reduced expression of CYP2A6 modulates the anti-tumor immunity by disrupting the equilibrium between 20-HETE and EETs (Jiang et al., 2021).

#### 3.3.2 AA metabolite LXA/B produced *via* LOX enzymes

In addition to the pro-inflammatory LTs produced from AA, 12-LOX, in conjunction with 15-LOX, plays a role in the synthesis of lipoxins such as LXA4 and LXB4 (Figure 2). In the liver, administration of LXA4 or treatment with BML-11, a lipoxin receptor agonist significantly improves hepatic injury and decreases fibrosis by reducing inflammatory cytokine release and attenuating hepatocyte apoptosis/necrosis in all liver injury models tested (Zhang et al., 2007; Xia et al., 2013; Zhou et al., 2013; El-Agamy et al., 2014; Zhang et al., 2015; Yan et al., 2016; Hu et al., 2017; Karaca et al., 2022). The effects of LXA4 are mediated *via* the renin angiotensin (RAS) system (Hu et al., 2017; Chen et al., 2019) and downregulation of NFκβ in hepatocytes and macrophages has been observed with LXA4 treatment (Kuang et al., 2016). In addition, LXA4 promotes apoptosis and inhibits cell proliferation and migration, and blocks EMT in HCC cells (Hao et al., 2011; Xu et al., 2018).

#### 3.3.3 Pro-resolving eicosanoids produced from n-3 PUFAs

Since the 3-series PGs and 5-series of LTs produced from EPA and DHA are low-inflammatory lipids compared to those produced from AA metabolism (Li et al., 1994; Whelan et al., 1997), a lower ratio of EPA/AA is suggested to be a clinical sign of inflammation (Ebright et al., 2022). In ob/ob mice, supplementation of *n*-3 PUFAs attenuates hepatic steatosis (Gonzalez-Periz et al., 2006; Gonzalez-Periz et al., 2009). In the liver cancer model where PTEN loss drives steatosis and cancer (Stiles et al., 2004; He et al., 2016; Jia et al., 2017; Chen et al., 2021b), supplementation with a *n*-3 PUF, EPA, significantly attenuates both NASH and cancer development (Ishii et al., 2009). Supplementation with fish oil, the major dietary source for EPA and DHA antagonizes the production of AA-derived eicosanoids (Li et al., 1994) and also significantly ameliorates biochemical parameters observed in HCC induced by DEN treatment (Metwally et al., 2011). Together with the observation that RvD inhibits FOXM1 expression in CAFs and represses EMT and cancer stemness (Sun et al., 2019), a potential role of resolving lipids in regulating cancer stemness through PTEN-PI3K signaling maybe proposed.

Attenuation of GPCR and cAMP mediated signaling is associated with the anti-tumor effects observed with EPA and DHA (Smith et al., 2006), suggesting the involvement of eicosanoids, oxylipins and their receptor signaling in these effects. During the progression of NAFLD/NASH in HFD models, levels of RvD1 and MaR1 significantly decreases with disease progression (Maciejewska et al., 2020). Lower circulating levels of MaR1 and RvD1 were also reported for NAFLD/NASH patients (Monserrat-Mesquida et al., 2020; Fang et al., 2021). RvD1, MaR1 as well as RvE1 are found to regulate lipid biosynthesis in hepatocytes (Jung et al., 2014; Rius et al., 2017; Jung et al., 2018; Oh et al., 2022). *In vivo*, treatment with these SPMs led to reduced expression of FASn and ACC-1 and lower levels of liver TG (Laiglesia et al., 2018; Rodriguez et al., 2020; Zeng et al., 2022). During liver injury, these SPMs display a hepatoprotective effect (Figure 4) *via* attenuating the inflammatory responses, inhibiting hepatocyte apoptosis and oxidative stress (Murakami et al., 2011; Zhang et al., 2015; Kuang et al., 2016; Wang et al., 2016; Li et al., 2020b; Zhang et al., 2020b; Soto et al., 2020; Tang et al., 2021; Hardesty et al., 2023). In cultured hepatocytes, RvD1 also inhibits hepatocyte proliferation and is implicated in attenuating cancer growth (Lu et al., 2018b). Consistently, both RvD and RvE prevent the progression to cancer (Kuang et al., 2016; Rodriguez et al., 2020) and MaR treatment mitigated fibrosis induced by DEN (Rodriguez et al., 2021). Again, the AKT-mTOR regulated autophagy in HSC contributes to the RvD inhibited fibrosis (Li et al., 2021). Together, these studies suggest RvD and RvE antagonize the protumor cell growth signals from the tumor microenvironment (Figure 4). Finally, in primary hepatocytes, MaR transcriptionally regulates FGF21 (Martinez-Fernandez et al., 2019), of which the function is mimicked by the recently approved liver fibrosis therapy efruxifermin (Tillman et al., 2022). Together, these studies suggest a role of resolvins in tumor microenvironment signaling.

## 4 Oxylipin and functions in liver macrophages

Chronic liver injury and accompanied inflammation establishes the liver microenvironment that permits and promotes cancer growth (Tu et al., 2022). Liver is known as an immunosuppressive organ as illustrated by the lower dose of immunosuppressive therapy needed for liver transplantation as compared with other organ transplantations (Huang et al., 2018). Liver resident macrophages, i.e. Kupffer cells play a critical role in this process as they inhibit cytotoxic T lymphocytes and induce T cell apoptosis (Huang et al., 2018). In healthy mouse livers, Kupffer cells develop this unique response by inducing the proliferation of select T regulatory cells, resulting in systemic immune suppression (Heymann et al., 2015). Cytokines produced by resident as well as infiltrating macrophages such as TNFα, TGF-β, IL-6 and IL-18 are highly associated with the development and progression of HCC (Hanahan and Weinberg, 2011; Del Campo et al., 2018; Tanwar et al., 2020). Consistently, increased macrophage activation/recruitment is a hallmark of liver cancer and implicated in a poor prognosis in patients (Hanahan and Weinberg, 2011; Debebe et al., 2017; Tu et al., 2022).

In the liver tumor adjacent tissues, COX-2 expression is highly expressed (Koga et al., 1999) and this increase is correlated with a shorter disease-free survival (Kondo et al., 1999). Later studies show that macrophage and mast cell populations are also higher in the tumor surrounding regions than within the tumors themselves, suggesting a role of COX-2 in modulating the immune microenvironment in addition to directly acting within the tumor cells (Cervello et al., 2005). Inhibition of COX-2 and downregulation of PGE2 with treatment of celecoxib/etoricoxib or loss of PGES1 leads to downregulation of IL-1β and TNFα, two microphage produced cytokines in the liver (Henkel et al., 2018; Ali et al., 2022), further indicate a role of COX2/PGE2 in macrophage function. In addition, the induction of 5-LOX was accredited to the stemness promoting function of CAF-programed myeloid-derived suppressor cells in iCCA (Lin et al., 2022). The induction of 5-LOX and elevated LTB4 or cysteinyl-LTs are associated with macrophage morphology and number changes in liver injury induced inflammation (Li et al., 2014; op den Winkel et al., 2013). Thus, alterations of bioactive lipid metabolism in the tumor microenvironment likely alters macrophage function and plays a role in liver cancer progression. In this section, we will review the literature exploring the production of bioactive lipids during liver injury and their functions on liver macrophages.

### 4.1 Macrophages produce oxylipins during liver disease progression

As with other macrophages, short term exposure of endotoxin dose-dependently induces release of prostanoids from the primary liver resident macrophages, Kupffer cells (Bowers et al., 1985; Tripp et al., 1988; Dieter et al., 1989; Peters et al., 1990; Horiuchi et al., 1992; Dieter et al., 2002; Bezugla et al., 2006). Compared to other macrophages, Kupffer cells in the liver are the most active at producing PGE2 (Wu et al., 1993). Both COX2 and PGES1 expressions are induced in liver macrophages 3–24 h after LPS treatment (Connor et al., 2013). In patients and animal models of steatotic liver disease, macrophages are also identified to be the source for the secreted PGE2 (Cao et al., 2022) and depletion of Kupffer cells led to reduced PGE2 production in the liver (Bai et al., 2010). Inhibiting COX2 in Kupffer cells also resulted in reduced TXA2 production in the liver (Pestel et al., 2002; Schade et al., 2002; Yokoyama et al., 2003), while depletion of Kupffer cells leads to reduced TXB2 production in perfusion extracts of livers (Oikawa et al., 2002). In multiple experimental settings, Kupffer cells are shown to be the primary source for TXA2 (Pestel et al., 2002; Schade et al., 2002; Steib et al., 2007). Media from cultured Kupffer cells from BDL mouse livers contains significantly higher concentrations of TXA2 than those from normal uninjured mice (Miller et al., 2007), indicating that the macrophage production of TXA2 can be induced during inflammatory response. These TXAs produced from Kupffer cells bind to their receptors, TP to induce T-cell activation and promote immune infiltration (Kabashima et al., 2003).

When COX2 activities are inhibited, Kupffer cells switch to produce LTB4 and 15-epi-LXA4 (Planaguma et al., 2002). Kupffer cells from injured livers also produce more LTs than PGE2 in response to phorbol ester or calcium ionophore treatment (Alric et al., 2000). Kupffer cells synthesize LTAs, and hepatocytes then convert these LTAs into LTCs (Sorgi et al., 2017). Hepatocytes readily uptake and can metabolize injected leukotrienes (Keppler et al., 1987; Leier et al., 1992). Supporting this, 5-LOX expression is primarily expressed in Kupffer cells and stellate cells, but to a lesser extent in hepatocytes themselves (Sala et al., 2010). In addition, studies depleting Kupffer cells suggest a role of Kupffer cells in 12- and 15-LOX regulated LT productions in the liver (Dragomir et al., 2011). When exposed to apoptotic cells, liver macrophages are also shown to express 15-LOX and produce immunosuppressive HETEs and HDEAs (Snodgrass et al., 2021), though spleen and bone marrow derived macrophages may be the major sources of the pro-resolving SPMs rather than Kupffer cells (Noureddine et al., 2022). As the bioactive lipids are important mediators for inflammatory functions, the amount and species produced by macrophages under specific disease conditions dictates the specific inflammatory responses.

### 4.2 The effects of bioactive lipids on kupffer function during liver disease progression

**FIGURE 5:**
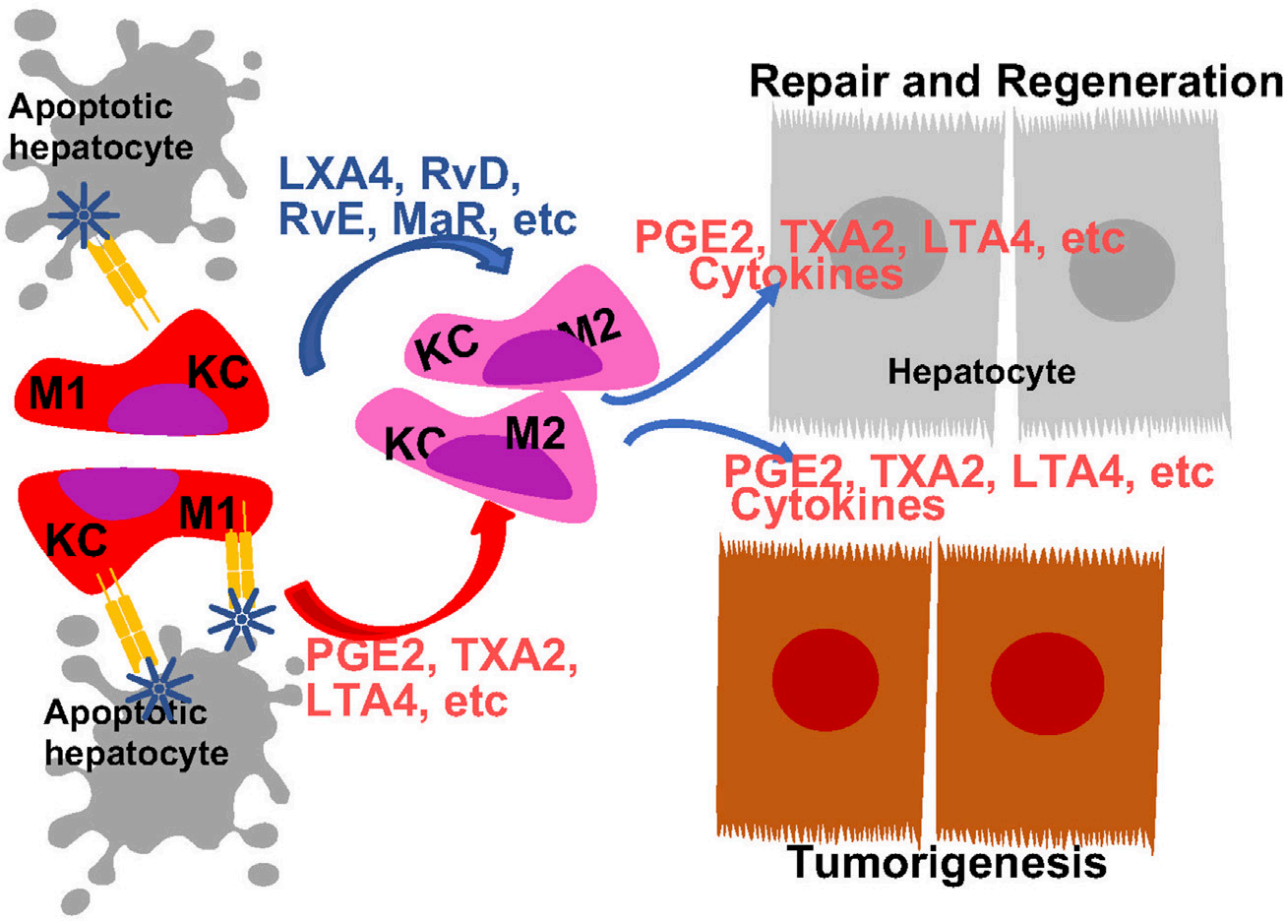
Regulation of Macrophage Polarity by Eicosanoids and Oxylipins and Its Role in Liver Disease Progression. Liver resident macrophages, Kupffer cells (KC) are the first responders to liver injury. In response to injury, they are programmed to produce M1 proinflammatory cytokines to orchestrated inflammatory response. Both eicosanoids with proinflammatory functions and pro-resolving eicosanoids induces macrophage polarization towards M2 phenotype. The M2 polarized macrophage also produces eicosanoids to promote tissue regeneration an tumor growth.

**FIGURE 6:**
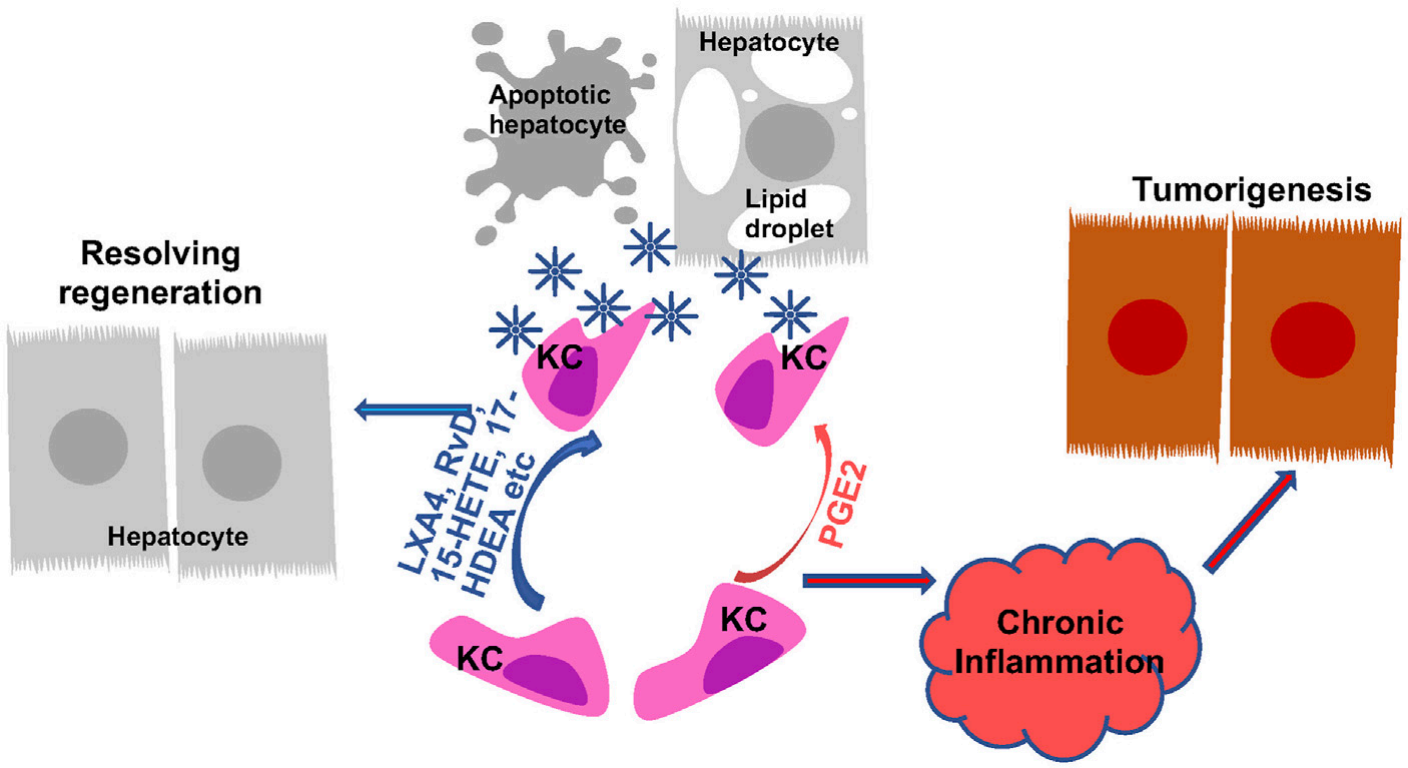
Regulation of Macrophage Phagocytosis by Eicosanoids and Oxylipins and Its Role in Liver Disease Progression. Kupffer cells (KC) are the primary phagocytic cells in the liver. Their phagocytic activities are induced in response to apoptotic cell debris, lipid particles and other signals. PGE2 and pro-resolving lipids both programs KCs to induce their phagocytosis abilities. The induced phagocytotic KCs promote resolution of inflammation, leading to regeneration from liver injury. When the phagocytotic activity of KCs are not induced or not sufficient to produce resolution, chronic inflammation establishes the tumor microenvironment to promote tumorigenesis.

Bioactive lipids also program liver macrophages during liver disease progression (Figures 5, 6). Consistently, treatment with inhibitors for 5-LOX and its activating protein (FLAP) lead to changes in morphology and apoptosis in Kupffer cells, resulting in macrophage depletion and decreased inflammation in the liver (Titos et al., 2003; Titos et al., 2005). Inhibition of leukotriene synthesis with lipoxygenase inhibitors (azelastine, ketotifen and AA861) attenuates ROS production from macrophages induced by liver injury (Shiratori et al., 1990). In addition, disrupting LTB4 receptor suppresses expression of EGF, VGEF and VGEF receptors in macrophages and plays a role in macrophage recruitment and liver injury induced by ischemia reperfusion (Ohkubo et al., 2013). On the other hand, PGE2 and TXA2 also attenuate liver macrophage recruitment and PGE2 treatment inhibits IL-1, IL-6 and ROS production from liver primary macrophages in a dose-dependent manner (Funaki et al., 1992; Kulkarni et al., 2021; Xun et al., 2021; Lan et al., 2022). PGE2 exerts its effects on the LPS-induced release of cytokines in rat liver macrophages *via* the EP2 and EP4 but not EP1 and EP3 receptors (Treffkorn et al., 2004; Lan et al., 2022).

The SPMs promote the anti-inflammatory and inflammation resolution responses of macrophages (Wall et al., 2010; Brennan et al., 2021). In sepsis mice, the EPA pre-conditioned adipose tissue mesenchymal stem cells normalize the morphology of liver macrophages (Silva et al., 2019). In HepG2 cells cultured with conditioned medium from activated macrophages, the macrophages stimulate hepatocyte proliferation is attenuated with treatment of LXA4 (Hao et al., 2011). Depleting KCs by liposome clodronate abrogates the effects of RvD1 on proinflammatory mediators in the injured livers (Kang and Lee, 2016), suggesting a role of macrophage in RvD1 regulated resolution of inflammation. RvD1 also markedly attenuates macrophage changes induced by IR and inhibited hypoxia-induced expression of IL-1β and IL-6 (Rius et al., 2014; Kang and Lee, 2016). The effects of RvD on liver macrophages also primes the caloric restriction on steatosis and cancer (Rius et al., 2014; Debebe et al., 2017).

#### 4.2.1 Macrophage polarization during liver disease progression

Macrophage functions can be defined by their polarization based on their cytokine production profiles in response to specific stimuli. The various degrees of polarization from M1 to M2 represent macrophage heterogeneity/reprogramming during the inflammatory responses. Tissues undergoing inflammatory response often harbor macrophages with both M1 and M2 polarizations. During liver injury, Kupffer cells are polarized to pro-inflammatory M1 phenotype in an effort to repair the tissue (Kang and Lee, 2016; Tu et al., 2020). Reprograming from M1 towards M2 phenotype is associated with resolution of inflammation and tissue regeneration. The bioactive lipids are implicated in this shifting of macrophage polarization from M1 towards M2 inflammatory phenotypes (Figure 5). This is evidenced by the altered macrophage polarization due to changes of dietary ratio of AA and EPA (Enos et al., 2015).

While often defined for their pro-inflammatory functions, eicosanoids also play a role in resolution of inflammation by promoting M2 polarization of macrophages. Liver macrophages from mice lacking PGES1 or treated with EP4 antagonist are polarized towards M1 inflammatory profiles (Nishizawa et al., 2018; Fang et al., 2021). In coculture systems using HCC cells, macrophages and T cells to mimic HCC microenvironment, M2 polarization is observed and found dependent on COX-2 expression (Xun et al., 2021). Thus, PGE2 production from macrophages appears to be associated with a M2 inflammatory state. These M2 polarized macrophages produce cytokines and growth factors that interacts with other cell types in the liver to drive the disease progression. For example, the M2 macrophages induces stellate cell (HSCs) autophagy to drive liver fibrosis (Cao et al., 2022). This process involves the production of PGE2 from the M2 macrophages and EP2 receptor that mediates the proliferation inhibition of the HSCs (Koide et al., 2004). Other proinflammatory eicosanoids have been shown to have opposite effects on HSCs (Pu et al., 2021; Li et al., 2023), though the involvement of macrophages were not explored. In the coculture system with macrophage, T-cells and hepatocytes, M2 polarized macrophages produce TGFβ to regulate T cell activity (Xun et al., 2021). Exhaustion of CD8^+^ T cell in this culture is caused by high COX-2-expressing HCC cell lines.

Consistent with an anti-inflammatory role of EPA produced eicosanoids, EPA-PC and EPA-PE reduce the elevated levels of serum TNF-alpha, IL-6 and MCP1 and attenuated macrophage infiltration in the liver (Wei et al., 2020). During liver injury, a proinflammatory condition is induced with decreasing M2 and increased M1 markers are observed in Kupffer cells (Kang and Lee, 2016). RvD attenuates these effects and leads to resolution of the proinflammatory conditions, while depletion of KCs by liposome clodronate abrogates this effects of RvD1 on proinflammatory mediators and macrophage polarization (Rius et al., 2014; Kang and Lee, 2016). Serving as a ligand for ROR, MaR1 also induces a M2 polarization in liver macrophages (Han et al., 2019). Together, these studies suggest both eicosanoids and pro-resolving oxylipins play roles in M2 macrophage polarization (Figure 5).

#### 4.2.2 Phagocytic functions of macrophages during liver disease progression

In addition to influencing macrophage polarization and cytokine production, the ability of macrophages to phagocytize apoptotic cells is also affected by eicosanoids. Efferocytosis, resulting from phagocytosis of engulfed apoptotic cells, particularly neutrophils, plays a key role in resolving underlying inflammation in liver disease. The unresolved inflammation and resulting chronic inflammation establish the immune tumor microenvironment for the development of HCC in the liver (Westbrook and Dusheiko, 2014; Fishbein et al., 2021; Heredia-Torres et al., 2022). While very little is known about the involvement of eicosanoids in efferocytosis of liver macrophages specifically, a role of eicosanoids in macrophages efferocytosis in general is well established (Thornton and Yin, 2021). In particular, SPMs are shown to be highly effective at promoting resolution of inflammation (Figure 6). Upon exposure to apoptotic cells, macrophages upregulate 5-LOX expression and enhances their ability to produce the pro-resolving SPMs including 15-HETE, 17-HDHA and RvD5 to participate in resolving inflammation (Snodgrass et al., 2021). The pro-resolving M2 macrophages prepared from human monocytes upregulate several resolvins include RvD, RvE and MaR and LXA4 and downregulate other pro-inflammatory eicosanoids including LTB4 to modulate their abilities to participate in efferocytosis (Dalli and Serhan, 2012). The pro-resolving lipoxins primarily exert their pro-resolving effects by binding to GPR32 on phagocytes and enhance their ability to phagocyte zymosan and apoptotic neutrophils (Krishnamoorthy et al., 2010). Due to the pro-resolving functions of LXA4 (Serhan et al., 1993), a stable LXA4 analogue, NAP1051 with a longer half-life has been developed (Dong et al., 2021). Like LXA4, this analogues inhibits neutrophil migration, and induce apoptosis of neutrophils, leading to a general resolving function.

During the phagocytic process, macrophages actively downregulate the production of cytokines including IL-1, IL-10, TNFα as well as LTC4 and TXB2 (Fadok et al., 1998). The production of PGE2, however, is increased in this process (Fadok et al., 1998). In several studies, blocking COX2 expression and PGE2 production is associated with reduced macrophage efferocytosis (Medeiros et al., 2009; Frasch et al., 2011; Salina et al., 2017; Sheppe and Edelmann, 2021; Ampomah et al., 2022). This function of PGE2 is thought to play a role in inflammation resolution during efferocytosis (Fadok et al., 1998; Takayama et al., 2002; Tang et al., 2012; Ampomah et al., 2022). Supporting this phagocytosis promoting role of PGE2, the phagocytosis ability of peritoneal and bone marrow derived macrophages is both attenuated in IBD mice carrying macrophage deletion of COX2 (Meriwether et al., 2022). In a zebra fish model, similar effects are observed (Loynes et al., 2018). However, here, PGE2 is shown to dose dependently drive neutrophilic inflammation resolution in the absence of macrophages (Loynes et al., 2018). Given the previous defined pro-inflammatory functions of PGE2, these effects of PGE2 on phagocytosis likely indicate a context-dependent role of PGE2 on inflammation and its resolution. The role of PGE2 in liver Kupffer cell phagocytosis and resolution of inflammation remain to be understood.

## 5 Forward and perspective

**FIGURE 7:**
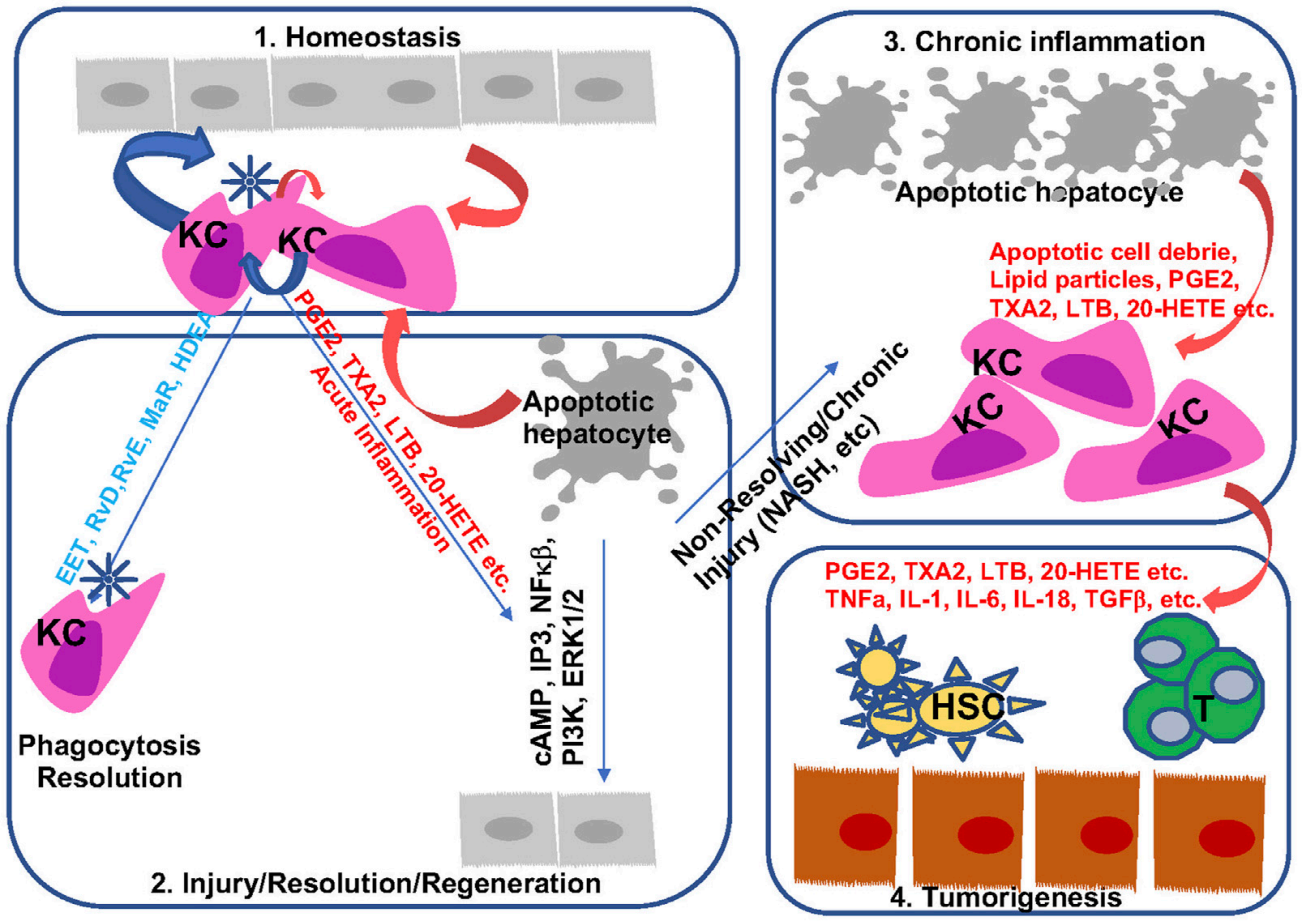
Bioactive lipids and progression of liver disease. During homeostasis, hepatocytes and macrophages produce a balanced levels of pro- and anti-inflammatory lipid species to maintain liver immune environment (Sung et al., 2021)**.** In response to acute injury, apoptotic cell debris alters macrophage polarization to produce both pro- and anti-inflammatory lipids. These lipids are involved in the resolution of inflammation as well as regeneration of hepatocytes (Singal et al., 2020). During chronic inflammation when sustained injury is present such as those presented with steatohepatitis, proinflammatory eicosanoids together with apoptotic cell debris sustain injury by polarizing macrophage towards a M2 phenotype (Sanyal et al., 2010). In addition, downregulation of resolvins permits chronic inflammation with reduced phagocytic activities from macrophages. The mitogenic signal from the proinflammatory eicosanoids and pro-tumor cytokines present a pro-tumor immune microenvironment to promote the progression from chronic inflammation to HCC (Kudo, 2017). They also interact with other cells (HSC an T cells as example) in the liver to regulate liver disease progression.

Inflammation is indispensable for the development and progression of liver disease from acute and chronic liver injury to metabolic liver disease to fibrosis and cancer. During this process, eicosanoids and other oxylipins are shown to regulate the balance of a pro-inflammatory vs anti-inflammatory conditions (Figure 7). In general, acute liver injury induces a pro-inflammatory conditions. AA-derived eicosanoids play a role in promoting this condition that include multiple inflammatory cell types while promoting regeneration *via* their mitogenic signal (Finetti et al., 2020). During chronic liver disease such as those involved in the development of NASH, fibrosis and HCC, eicosanoids with pro-inflammatory functions are maintained. Paradoxically, these pro-inflammatory eicosanoids also play a role in resolution of inflammation by regulating macrophage polarity and phagocytotic function. Together with SPMs, the pro-inflammatory eicosanoids such as PGE2 induces macrophage polarization towards a M2 phenotype. During this progression, the lack of SPMs may have attenuated the ability of macrophages to perform its phagocytotic function for full resolution of inflammation, resulting in accumulation of M2 polarized TAMs in the inflammatory tumor microenvironment. While this review is focused on the changes of eicosanoids during liver disease progression and their functions in macrophages, the effect of these lipids on other cells types such as HSCs, T-cells, neutrophils also play a role in establishing the inflammatory tumor microenvironment (Figure 7). Eicosanoids, particularly PGEs have been shown to regulate the function of these other cell types (Zhang et al., 2010; Hao et al., 2011; Brudvik et al., 2012; Xun et al., 2021). However, as the lipid species, their effects on different cell types, and the reaction of the different cell types change significantly throughout the stages of liver disease, a more clearly defined stage/disease condition related profile of lipid species and cellular heterogeneity needs to be defined prior to fully understand how they interact with each other. Such effort has been put forth in recent years as illustrated by studies showing that HSC produced PGE2 in NASH promote tumor tumor growth (Loo et al., 2017).

The M1/M2 polarity is often used in defining the role of these bioactive lipids on macrophage function. However, the liver macrophages are highly heterogenous comparing to other tissue types. A simple M1 vs M2 phenotyping does not distinguish the diverse macrophage types in the liver and both M1 and M2 phenotypes are stimulated simultaneously in different state of the disease progression. For example, the liver resident macrophage KCs maintain liver homeostasis by inhibiting the cytotoxic T cells (anti-inflammatory) while presenting the ability to eliminate antigens through phagocytotic functions (resolution) and release IL-6 (pro-inflammatory) when treated with LPS. In this scenario, KCs secret PGE2 and 15-deoxy-delta12,14-PGJ2 (15 days-PGJ2) to suppress effector T cells induced by other APCs (You et al., 2008). Thus, while PGE2 is associated with a traditional M2 polarizing cytokine phenotype, this view to define their function towards inflammation and their roles in liver disease progression is limited. Recent single cell RNA-seq studies have started to define the macrophage diversity under different conditions (MacParland et al., 2018; Ramachandran et al., 2019; Bleriot et al., 2021). A more precise view of the diverse macrophage populations (as well as other cell types), and their eicosanoids profile needs to be defined together with their functions during liver disease progression. In either case, the current literatures established the roles of both pro- and anti-inflammatory bioactive lipids in liver disease progression and macrophage function. However, significant knowledge gap needs to be filled to fully understand how they contribute to and their regulation during liver disease progression and cancer development.
